# Recent Development of Ruminant Vaccine Against Viral Diseases

**DOI:** 10.3389/fvets.2021.697194

**Published:** 2021-11-03

**Authors:** Sk Mohiuddin Choudhury, XuSheng Ma, Wen Dang, YuanYuan Li, HaiXue Zheng

**Affiliations:** ^1^State Key Laboratory of Veterinary Etiological Biology, National Foot and Mouth Disease Reference Laboratory, Key Laboratory of Animal Virology of Ministry of Agriculture, Lanzhou Veterinary Research Institute, Chinese Academy of Agricultural Sciences, Lanzhou, China; ^2^Gansu Agricultural University, Lanzhou, China

**Keywords:** ruminant viral vaccine, development of vaccine, attenuated vaccines, DNA vaccines, subunit vaccines, inactivated vaccine, innate immunity and adaptive immunity, next generation vaccine technologies

## Abstract

Pathogens of viral origin produce a large variety of infectious diseases in livestock. It is essential to establish the best practices in animal care and an efficient way to stop and prevent infectious diseases that impact animal husbandry. So far, the greatest way to combat the disease is to adopt a vaccine policy. In the fight against infectious diseases, vaccines are very popular. Vaccination's fundamental concept is to utilize particular antigens, either endogenous or exogenous to induce immunity against the antigens or cells. In light of how past emerging and reemerging infectious diseases and pandemics were handled, examining the vaccination methods and technological platforms utilized for the animals may provide some useful insights. New vaccine manufacturing methods have evolved because of developments in technology and medicine and our broad knowledge of immunology, molecular biology, microbiology, and biochemistry, among other basic science disciplines. Genetic engineering, proteomics, and other advanced technologies have aided in implementing novel vaccine theories, resulting in the discovery of new ruminant vaccines and the improvement of existing ones. Subunit vaccines, recombinant vaccines, DNA vaccines, and vectored vaccines are increasingly gaining scientific and public attention as the next generation of vaccines and are being seen as viable replacements to conventional vaccines. The current review looks at the effects and implications of recent ruminant vaccine advances in terms of evolving microbiology, immunology, and molecular biology.

## Introduction

Vaccinology was founded with Edward Jenner's discovery of the smallpox vaccine, permanently transforming the history of medicine. He discovered that immunizing against a virus that is less virulent yet antigenically related (Cowpox virus) protects against a virus that is more virulent (smallpox virus) (1). The first century and a half since Jenner's discovery was mostly devoted to the Development and understanding of scientific fundamentals. Vaccinology's marvels have been shaped over the past five decades (1). The global eradication of smallpox, as well as major reductions of other viral diseases such as polio, measles, mumps, and rubella, show that vaccination is the most practicable and cost-effective tool for detecting, managing, and eradicating infectious diseases (2). The usage of the “Plowright” vaccine, for example, is generally thought to have been crucial in virtually eradicating the rinderpest virus from the globe. The Kabete O strain was passaged 90 times in tissue culture to establish this attenuated vaccine (3, 4).

Today's most vaccines on the market are either inactivated (killed) or live attenuated (weakened) (5). Many significant veterinary diseases have been effectively addressed using such methods. Both methods, though, have their own set of shortcomings and future issues. Vaccines that have been inactivated must be entirely safe and non-infectious (6). Incomplete inactivation has been blamed in the past for outbreaks in the area. Such issues should not happen if the production method utilized more accurate inactivates, inactivation processes, and innocuity checking. Furthermore, since the production of such vaccinations necessitates the culture of vast quantities of the infectious agent, there is a risk to both the workers and the community (7). Vaccines made in embryos, tissue culture, or culture medium can produce unwanted “foreign” proteins that may reduce immunogenicity or cause allergic reactions (8). Finally, inactivated vaccines' presentation style and the quality of the immune response they can evoke are restricted (7). The reaction to vaccination may be small and short-lived, necessitating the use of adjuvants or immunostimulants to improve overall immunogenicity and efficacy. Attenuated vaccinations must be strictly controlled and specified in order to have the desired level of protective immunity without having severe disease effects in the host animal. Also a remote possibility that the attenuated antigen will revert to full virulence necessitates meticulous virulence defense checks (9). Furthermore, other infectious agents could be added to the vaccine antigen culture, which may contribute to unintended side effects as the vaccine is used in the field. For these and other reasons, scientists are progressively focused on new vaccine technologies, including preventive efficacy, production expense, and whether the infectious agent can be produced *in vitro*. These vaccine technologies include split-product, subunit, isolated protein, peptide, marker vaccine, live vector, and nucleic acid approaches.

Vaccines for ruminants are used to accomplish a variety of objectives. The key objectives are to provide cost-effective methods for preventing and controlling infectious diseases in cattle, improving animal welfare, and reducing the yield of food animals (10). The widespread vaccination of wildlife, on the other hand, has been remarkably regarded as a way of avoiding the transmission of zoonoses (11). Furthermore, as a consequence of widespread vaccine campaigns, the intake of numerous ruminant medications has declined dramatically, reducing their environmental influence, side effects, and contaminants in food animal goods. To summarize, ruminant vaccinations have greatly increased human well-being while still bolstering animal welfare. The majority of newly licensed veterinary vaccines are either killed or live vaccines that have been modified. Despite the fact that widespread use of these vaccines has greatly enhanced ruminant and public well-being around the world, they are not without drawbacks and are far from perfect. Traditional vaccinations are usually expensive to produce (inactivated Vaccines), need adjuvants (inactivated Vaccines) and several doses (live attenuated and inactivated vaccine) to cause sufficient immunity, interfere with maternal antibodies (live attenuated, inactivated, subunit vaccines), and have little or no protection for newborns (12). Toxoid vaccines are created using pathogens toxins. They provide protection against the disease rather than the infection itself. Toxoids evoke a consistent humoral immune response but little to no cell-mediated immunity (13, 14). Toxoid vaccinations, in contrast to attenuated viral vaccines, do not often last for long periods of time. Therefore, like other kinds of immunizations, toxoid vaccines may need booster injections for continuing protection. Depending on risk factors, revaccination (booster) may be needed several times within a single year (15–17). They may also trigger negative side effects as a result of undesirable elements, such as endotoxins (18). Due to all of these drawbacks, continuous researches are essential for the development of vaccines and vaccination. The goal of the study is to describe the existing methodologies used to construct traditional ruminant vaccine, as well as the next generation approaches for developing these vaccines. This article would not go into great depth on vaccinations and vaccination in the global animal health market. Still, it will aim to concentrate on ruminant animal health of concern.

## Host Defense Mechanism

Vaccination seeks to activate the immune system in such a way that the host can develop an efficient (and potentially long-lasting) memory immune response that can track and eventually eliminate the pathogen once it has invaded the body (19). This may be accomplished by administering an antigenic stimulation (vaccine). An effective vaccine must be seen as a non-self agent that, ideally, activates innate immune responses before “instructing” adaptive and memory responses. Unlike innate immunity, adaptive immunity recognizes foreign antigens in a very precise way (20). Adaptive immunity is mostly humoral and cell-mediated (14). Extracellular infections may be avoided by strengthening humoral immunity. Extracellular pathogens, which exist and reproduce outside host cells in alimentary, urogenital, and respiratory tracts, are avoided by host monocytes, neutrophils, and macrophages in the process of being phagocytosed and killed. It is the generation of particular antibodies and activation of the complement system that are responsible for the primary effector function of host immunity to regulate and remove the external infection. An intracellular defense against pathogens is a complicated process that depends on powerful cell-mediated immune responses (21). During infection, pathogens normally remain in the host cell and multiply within it. The immune system may interfere with any of these phases, which may prevent the illness. Inhibiting the adhesion and entrance of intracellular infections may be the most efficient way of preventing disease (22, 23). An effective humoral immunity may prevent the host cells without Fc receptors from being infected by pathogens (24). Moreover, antibodies may bind to pathogens and make them easier for phagocytes carrying Fc receptors to take up. The internalization process that is performed by phagocytes is detrimental to most infections and leads to the breakdown of engulfed organisms (25, 26). Following the host cell entrance, humoral immunity is unlikely to be effective against the pathogen, therefore a robust cell-mediated immunity is needed to constrain and eliminate the internalized pathogen. Inside the host cell, intracellular pathogens are either held in membrane-bound vesicles (phagosomes) or the cytoplasm (27).

**Figure 1:**
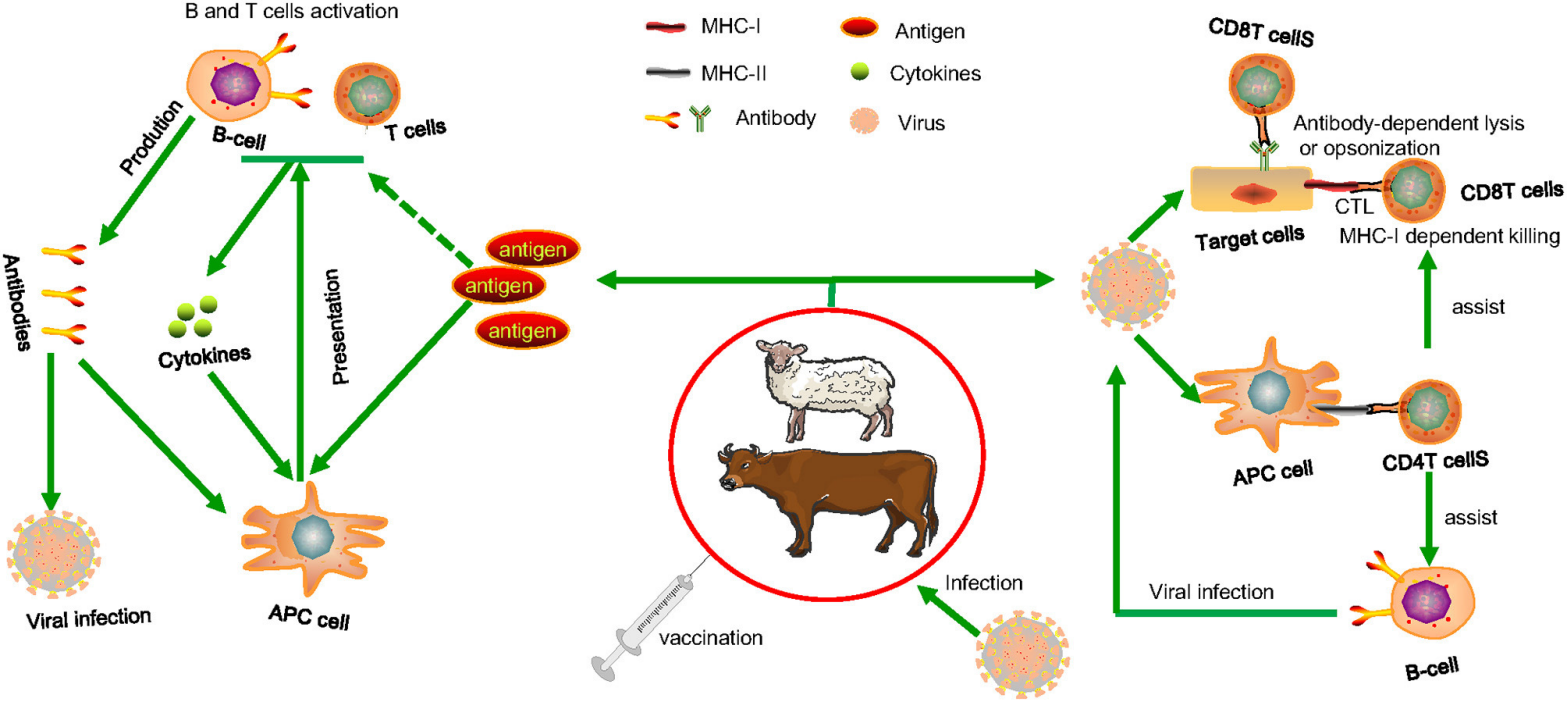
The cellular cooperation in the immune response. Following vaccination, advanced phagocytes present the processed antigens to naive B or T cells, which can be activated if co-stimulatory signals are produced (derived from the interaction of PAMPs with cellular PRRs). When lymphocytes become stimulated, they release soluble mediators and antibodies that cause inflammatory responses. In this simplified illustration, immune systems that may protect animals from invading viral infections are illustrated. CTL that identifies pathogen-derived epitopes presented in combination with MHC class I on infected cells or antibody-dependent lysis or opsonization of infected cells expressing pathogen molecules. Extracellular and intracellular pathogens on their way to infect other cells, can be attacked by specific circulating antibodies and killed by lysis or agglutination or phagocytosed by macrophages and neutrophils. Assistance is required from pathogen specific CD4 helper T cells both for antibody and CTL induction and that are activated after interaction with pathogen-derived epitopes presented in combination with MHC class II molecules on the surface of MHC class II^+^ antigen-presenting cells. When pathogens infect antigen-presenting cells, they can be killed directly by CD4 T cells and CD8 CTL through the induction of mediators such as interferon gamma (IFN-γ), reactive oxygen and nitrogen species, and indoleamine 2,3-dioxygenase. Pathogen toxins can be neutralized by circulating antibodies, resulting in decreased infection-related clinical symptoms.

As we see in Figure 1, Antigenic determinants can be presented to näive (B and T) lymphocytes by infected cells or specialized phagocytic cells (antigen-presenting cells or APCs, such as macrophages and dendritic cells) (28, 29). As a general rule, antigens from vesicular intracellular pathogens are processed and presented in the context of major histocompatibility complex (MHC) class II molecules to activate näive or reactivate memory CD4+ T-cells (30, 31). Antigens of cytoplasmic intracellular pathogens are processed and delivered to CD8+ T-cells through the class I processing and presentation pathway (32). Although it is known that antigens that are produced outside of cells (exogenous) may be presented through the class I pathway, it has been shown that this may be achieved by linking antigens to various peptides, thus influencing the presentation of an antigen by a certain MHC molecule (33). CD4+ T-cells are powerful effectors, and CD4 failure is connected to increased vulnerability to different diseases. CD4+ T-cells play a crucial role in both the induction (antigen recognition and T-cell activation) and effector stages (cytokine production and cytosolic activity) of the immune response (34). Additionally, they offer assistance with pathogen-specific B-cell cloning and differentiation, and they have a large impact on the generation of antibodies by B-cells. Additionally, CD4 assistance is required for CTL (cytotoxic T-lymphocyte) activity induction and in particular for maintaining CD8 T-cell responses (35). The presence of virus-specific cytotoxic T cells is widely acknowledged as being necessary for the immune system to manage viral infection (CTL) (36). Dendritic cells and other professional antigen presentation cells activate CTL by processing viral proteins that are generated endogenously or taken up from infected cells through apoptosis (crosspriming) (37). Following the growth of a viral clone, the virus-specific CTL may begin killing infected cells by using perforin- and/or Fas-dependent pathways, thereby halting further viral particle creation. The death of the infected cells is completed in <4 h, after the presentation of viral peptides on the MHC class I molecules that is seen on the infected cell surface (38–40). This assault happens in conjunction with CTL's production of cytokines and chemokines that are antiviral. Again, in cellular-mediated immune responses to foreign antigens, CD8+ T cells play a crucial role (41). *In vitro*, it was shown that co-stimulating pure CD8+ T cells promotes *de novo* CD4 molecule production and that ligation of CD4 on this cell type regulates CD8+ T cell activity (42–44). CD4 expression on CD8 T lymphocytes influences cytotoxic T lymphocyte activity and is necessary for effective cell-mediated immunity against viruses and alloantigens *in vivo* (45–47).

Pathogen-associated molecular patterns (PAMPs) bind to intracellular PRRs (Pattern recognition receptors) and trigger phagocytic cells by inducing NFκB-mediated gene transcription of many co-stimulatory molecules, pro-inflammatory cytokines, and chemokines, as well as IRF-mediated gene transcription of type-I interferons (IFNs) and other cytokines such as IL-1β and TNF-α (48–50). NK cells express functional TLRs specifically for detecting viral PAMPs (51). IFN-γ, which is secreted by activated lymphocytes such as CD4 T helper cell type-1 (Th1) cells, CD8 cytotoxic T cells, natural killer (NK) cells, natural killer T (NKT) cells (52) can increase macrophage phagocytic activity and antigen presentation through mature dendritic cells (DCs), which are an important part of the innate-adaptive immunity bridging mechanism (53). DCs' capacity to signal naive lymphocytes can determine whether or not these cells are involved in fighting the virus. Vaccines based on attenuated viruses or replicating live virus vectors take advantage of this point rather than vaccines focused on inert antigens. The effectiveness and severity of adaptive responses are greatly improved when innate immune responses are triggered (inactivated virus or subunit vaccines) (29). Owing to their vital role in regulating the immune response, antigen targeting of dendritic cells, or APCs, has recently become a major priority for specific immune stimulation to improve vaccine efficacy and other immunotherapy forms (54, 55). When naive lymphocytes interact with DCs, clonal expansion of B and T cells capable of recognizing the same antigen arises, the immune response becomes more specific (56). As a result of the vaccine activation, a pool of advanced lymphocytes including memory and effector cells will be expanded. In response to infection and viral antigen encounters, the secondary response will be greatly improved, gradually contributing to protection and long-term immunity through specific effector and memory cells (14). B cells choose antigen experienced CD4 T cells to become memory and start an organized genetic program that preserves memory CD4 T cells throughout life of the individual (57). B cells collect less antigens during the clearance of an infection, and that makes previously antigen-exposed CD4 T cells become dormant. Without antigens, the resting state with minimal energy expenditure and multiplication keeps memory CD4 T cells alive in mice almost indefinitely (58). Low levels of antigen presentation may be a critical strategy for regulating CD4 memory T cell long-term survival and preventing cross-reactivity to autoantigens, and hence autoimmunity (59). The issue of precision is vital to a vaccine's efficacy. It can be resolved by choosing a suitable antigen fraction, whole antigen, or antigens of choice, as well as remembering the memory lymphocyte pools developed during the primary responses after vaccination (60). Determining the correlates of protective immunity following infection, such as related epitopes that elicit neutralizing antibodies and primary T-cell epitopes liable for helper or cytotoxic roles, is one of the logical methods for vaccine design (61, 62). This knowledge would ideally come from studies on the pathogenesis of viral infection in the target species for which the vaccine is being developed. These experiments, on the other hand, are much more complex to conduct than those involving experimental animals such as rodents (mainly due to the genetic diversity of the outbred species, the lack of reagents and markers for cell phenotype characterization, and the limitation in the number of animals used for experimentation). However, in some cases, the pathogenesis of other animal models of disease (primarily rodents) is sufficiently similar to that of the target species to include valuable information about protective immune mechanisms. Following the accumulation of data gathered over decades of viral research, it is apparent that successful immuno-prophylaxis can be accomplished by triggering an immune response against surface antigens expressed on virions and virus-infected cells for viruses with less complicated pathogenesis. In order to produce an efficient immune response to a pathogenic agent or an immunization, both the innate and adaptive immune subsystems are required. Furthermore, successful vaccinations must produce effector cells for the present infection and memory cells for future infections with the pathogenic agent, resulting in long-term activation of both the humoral and cell-mediated arms of the adaptive system as in most viral infections induction of both humoral and cellular immunity is important for protection (14, 63). Some viruses (such as poxvirus, herpesvirus, and lentivirus) have more complicated pathogenesis (i.e., persistence activation, replication in privileged immune tissues, immune escape processes, and recruitment of hazardous host immune responses) and need a vaccine that elicits specific T-cell responses in addition to neutralizing antibodies (29).

## Ruminant Viral Vaccines

While in their historic function in agricultural research and education, ruminants are considered to be essential. These ruminants are currently also utilized in investigations in molecular biology, genetic engineering, and biotechnology for applications in fundamental science and agricultural research and therapeutic usage. Public concern and curiosity in the welfare of these species and the biology and behavior of the animals have persisted and are reflected in updated husbandry and management techniques. But, several viruses like Foot and mouth disease virus (FMDV), Peste des petits ruminants virus (PPRV), Bovine viral diarrhea virus (BVDV), Bluetongue virus (BTV), Bovine herpesvirus type 1 (BHV-1), Capripox virus, etc. cause fatal diseases in ruminants having a great negative impact on both socio and economic condition. When there are no broad-spectrum antiviral pharmaceuticals usable, the only methods for avoiding or managing virus infections are vaccination and hygienic measures to reduce exposure. Some of OIE's notifiable diseases and their traditional vaccines are Live attenuated for BTV, BVDV, LSDV, PPRV, SPV (64–71) and inactivated for FMDV, BVDV, SPV (67, 70–72).

**Figure 2:**
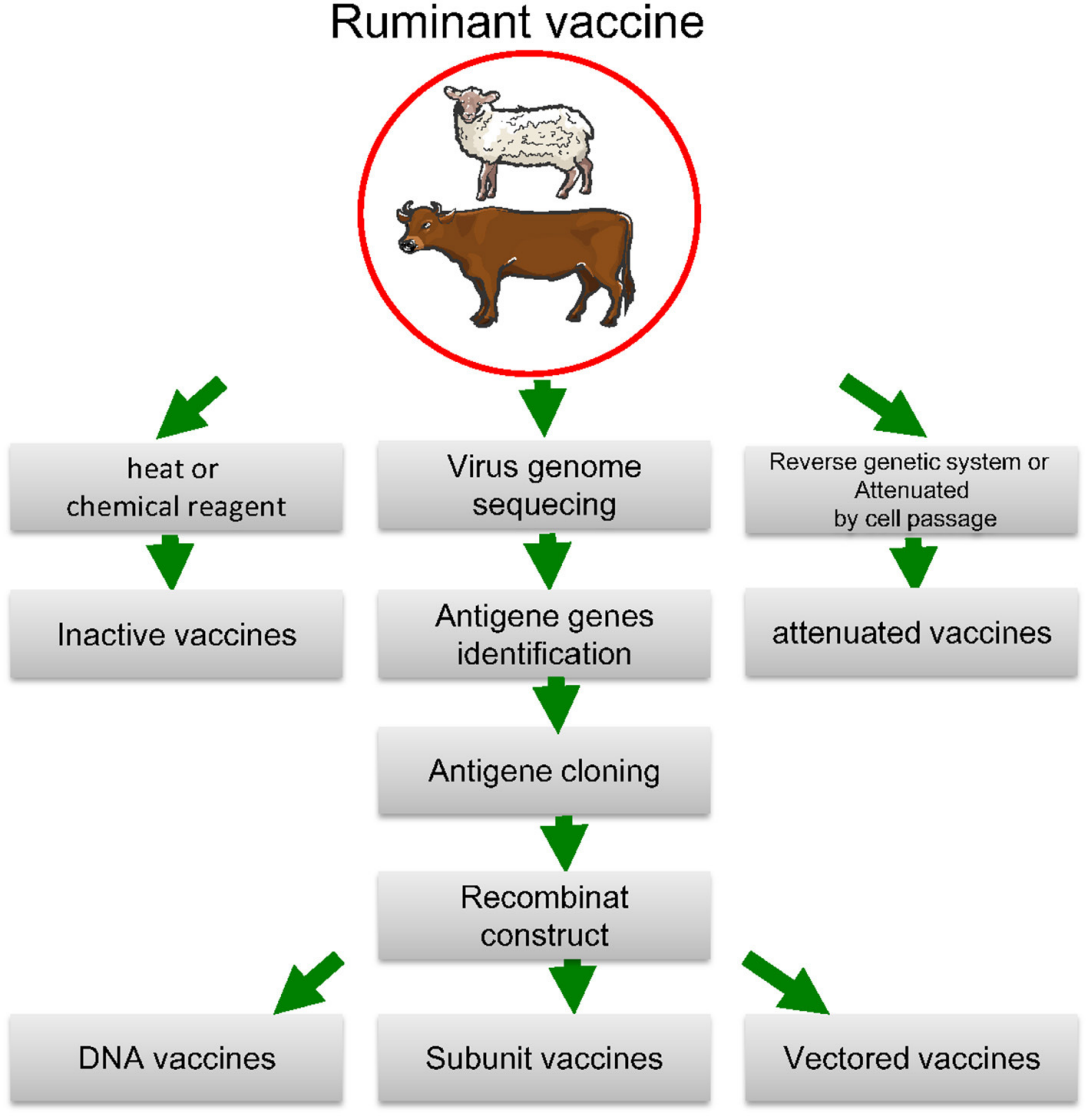
Biotechnological approaches to vaccine development. The antigen-coding gene is isolated and either expressed and extracted from a protein-production device or directly expressed by the vaccine receiver after injecting an engineered plasmid or a live vector. To extend the immune response, prime-boost techniques use a variety of antigen distribution mechanisms. The method with reverse genetics Attenuated vaccines are made with cell passage, whereas inactive vaccines are made with heat or chemical reagent.

Viruses (particularly RNA viruses) are extremely variable consisting of large numbers of variant genomes and through mutation they alter the nucleotide sequence of the genome. Viral replication constantly produces mutants, and their frequency of occurrence changes as replication progresses (73). One of the example of this issue is Sars-CoV infection (74). Viruses evolved several techniques to protect infected cells against CTL (cytotoxic T-lymphocyte) assault. Interference with the peptide-presenting pathway and viral epitope mutation are among the reasons for this issue (75). Viruses with multiple serotypes are responsible for several viral infections (e.g., FMD virus, bluetongue virus, and influenza viruses). As a consequence, many existing virus vaccines are often unable to cope with the most current strains in the region, necessitating the Development of new ones based on field strains that have recently caused outbreaks. For decades, the animal health industry has created a range of standardized live and inactivated virus vaccines that have been used in routine vaccine procedures for pets and livestock. The industry is seeing an influx of rationally formulated and subunit vaccines, and this segment will concentrate on these “second-generation” viral vaccines (summarized in Figure 2 and Table 1).

**Table 1:** OIE's notifiable disease and licensed vaccine.

**Diseases**	**Virus acronym**	**Host**	**Available vaccine**	**References**
Bluetongue	BTV	Cattle, Sheep, Goats, Buffalo, Deer	Live Attenuated	(66)
Foot and mouth diseases	FMDV	Cloven-hoofed animal	Inactivated (BEI)	(72)
Bovine viral diarrhea	BVDV	Cattle	Inactivated/Attenuated	(67, 69)
Lumpy skin disease	LSDV	Cattle	Attenuated	(68)
Infection with pestedes petis ruminants virus	PPRV	Sheep/Goat	Attenuated/Recombinant capripoxvirus	(64, 65)
Sheep pox and Goat pox	SPV	Sheep/Goat	Inactivated/attenuated	(70, 71)

## Live-Attenuated Ruminant Vaccine

Similar to the first human smallpox vaccination, certain live veterinary virus vaccinations induce minor infections of live cells from non-target hosts or are attenuated by passage across several cell line cultures or chicken embryos (eggs) (76). Random mutations are often used to establish attenuated viral strains, which are then chosen for decreased virulence (77). These vaccines can multiply and induce cellular and humoral immunity without using an adjuvant since the live organism can also infect target cells (78). Live drugs often benefit from being easy to administer, whether in drinking water, intranasally, intraocularly, or otherwise. They do, however, carry the possibility of latent virulence and reversion to pathogenic wild forms, as well as being a possible cause of pollution in the ecosystem. Although current regulatory systems demand data to assure these matters, challenges may emerge in the sector. Live virus vaccinations were crucial in the effective prevention and eradication of diseases (79).

Many of the Peste des petits ruminants (PPR) vaccine virus genes have attenuating mutations, but none are sufficiently debilitating to trigger intense pressure for reversion. The PPR vaccine is thought to be relatively safe, with no immunosuppressive impact on the host (80–82). While single point mutations in the polymerase gene have been shown to trigger sound attenuations, the high incidence of random mutations in RNA viruses raises the likelihood of reversion to virulence. As a result, various attenuating mutations spread across the genome are likely to be needed for safe live viral vaccines (83–85).

The bovine herpesvirus type 1 (BHV-1) causes contagious bovine rhinotracheitis (IBR), infectious pustular vulvovaginitis (IPV), and infectious balanoposthitis (IBP), as well as conjunctivitis, encephalomyelitis, mastitis, enteritis, and miscarriage, and vaccination is an essential part of preventive and eradication programs (86). In multiple herpesviruses, the thymidine kinase (TK) gene has been related to virulence, and deletion or insertion of the TK gene results in a stable, attenuated mutant. TK-BHV-1 vaccines, on the other hand, have been linked to dexamethasone-induced lag and reactivation. Attenuated vaccine strains have been generated by deleting BHV-1 glycoproteins such as gB, gC, gD, gE, gG, and gI, which are responsible for attachment, penetration, and cell-to-cell contact (87). It was discovered that knocking out both TK and gC protectagainst infection with wild-type BHV-1, and that a single glycoprotein gG, gI, and gE, as well as a double mutant of gI/gE, have attenuated the effect of a single glycoprotein gG, gI, and gE. Due to the poor immunogenicity of gI and gI/gE deleted mutants, only gG and gE deleted mutants have been proposed as vaccine candidates (88–90). In another research, the safety of the BHV-1 mutant with a deletion in glycoprotein E gene in calves was demonstrated. Intranasal inoculation of glycoprotein E gene deleted BHV-1 resulted in a 100-fold reduction in viral replication, shorter virus shedding, and overall reduced virulence, with no impact on neutralizing antibody production, according to immunogenicity findings (89, 91). A vaccine candidate with multiple deleted genes was evaluated for safety and efficacy (glycoproteins E and G and US2). Live-attenuated marker vaccines that differentiate between vaccinated and naturally infected animals have been developed using gene deletion techniques (92, 93). BVDV is a ruminant pestivirus that is very widespread. Subclinical manifestations, malnutrition, immunosuppression and leukopenia, congenital anomalies, infertility, and digestive tract erosions are among the risks (mucosal disease) (94). As a result, BVDV causes significant economic losses to the cattle industry by lowering reproductive success and milk yield and raising the prevalence of other infectious diseases and mortality (94). Vaccination has been commonly used in several BVDV surveillance programs in several nations (67). Live vaccinations have the potential to trigger transplacental diseases in pregnant animals and can even have immunosuppressive properties (69, 95–98).

## Inactivated or Killed Ruminant Vaccine

In comparison to live vaccines, inactivated or killed virus vaccines are generally more stable and may not be subject to virulence reversion; nevertheless, their inability to enter cells and cause cytotoxic T cells makes them less immunogenic (14, 99). As a result, strong adjuvants and multiple treatments are normally necessary to achieve the desired level of protection, even though they are usually mainly effective in treating clinical signs rather than infection (100, 101). Inactivated adjuvanted vaccinations are often more prone to induce inflammatory illnesses, allergic conditions, and sarcomas at the injection sites. Heat or chemicals are widely used to inactivate viruses (e.g., formaldehyde, thiomersal, ethylene oxide, and propriolactone) (102). These vaccines are more costly to produce due to higher manufacturing costs and the need for adjuvants. Inactivated viral vaccines for a wide range of viral diseases have been available for several decades and are still being developed for some recently emergent diseases (103). The creation of improved adjuvanted formulations to counteract maternal antibodies' effects on young animals has been the subject of much recent research in this field (104). Inactivated vaccines for various infectious diseases must be updated regularly to ensure that they include the correct serotypes (105).

The use of cross-linking agents (e.g., formaldehyde, glutaraldehyde, aldrithiol, or 2,2^/^-dithiodipyridine) for vaccine production is plagued by two primary disadvantages. The first is the possibility of aggregation, which may result in antigenic epitope disruption or alteration, potentially accounting for the vaccines' decreased immunogenicity, which typically requires two or three booster doses to maintain sufficient and long-lasting levels of protective immunity (106, 107). Another concern is the risk of insufficient inactivation, which could lead to disease exacerbation if partially (or suboptimal) induced immunity cooperates with infectivity through pathways such as antibody-dependent enhancement (ADE) (108). Virus complexed with non-neutralizing antibodies will attack monocytes or macrophages (cells with Fc-receptors) in this case, a process close to those seen in dengue virus infections (109). Finally, inactivated vaccinations face the challenge of solving the distinction between sick and vaccinated animals without interfering with surveillance diagnostics. Although formaldehyde primarily modifies proteins, propiolactone (BPL) and binary ethylenimine (BEI) primarily change DNA or RNA so that BPL can retain high immunogenicity throughout virus inactivation (110). However, certain amino acids, such as cysteine, methionine, and histidine, have been documented to trigger BPL to respond so that these protein modifications can influence BPL vaccines' immunogenicity (111). On the other hand, BEI has been also found to bind to proteins (112). This substance is widely used in the formulation of vaccines to inactivate the virus that causes foot and mouth disease (FMDV) (72, 113, 114). About this, inactivated vaccines remain a popular type of vaccine development (for both human and veterinary use), thanks to the efficacy of adjuvants (primarily aluminum salts) in vaccine formulations, which help to overcome the key problem of reduced immunity (115). In reality, other inactivation techniques, such as hydrogen peroxide or protonating compounds like diethylpyrocarbonate (DEPC), can profit from this technology (29). Hydrogen peroxide can inactivate all DNA and RNA viruses (vaccinia virus) while causing minimal disruption to the antigenic structure, reducing immunogenicity (116). Surprisingly, this inactivation method enabled vaccines to elicit humoral (neutralizing antibodies) and cellular immune responses targeted at CD8+ cytotoxic T-cells (117). The infectivity and pathogenicity of the vesicular stomatitis virus (VSV) was said to be eliminated when a histidine-protonating agent like DEPC is used (118). Despite advances in a variety of innovations for improving immune responses, the traditional inactivation approach is still widely used to develop most vaccines for ruminant use, in part because producers must closely balance the costs of adapting their current production methods to new technologies with expected profits. Other popular physical inactivation techniques necessitate exposure to various types of radiation, such as microwave, chemical, or ionizing radiation. UV radiation is one of the most often used techniques in the manufacture of human vaccines (119–121).

## Subunit Vaccines and Virus-Like Particles (VLPs)

Subunit vaccines include a component of the target pathogen which evoke an immune reaction that is unique to that section (122). While baculovirus-expressed Rinderpest virus (RPV) “H” and “F” proteins were used as antigens for subunit vaccines, they did not provide protection in cattle against virulent RPV despite eliciting a robust neutralizing antibody response (123). However, virulent virus challenge protection was reached when baculovirus-expressed H protein was inserted into immunostimulating complexes (ISCOMs) (124). Given that ISCOMs are considered to elicit a cell-mediated immune response, it's possible that the cell-mediated immune response is a key factor in triggering a protective immune response against morbilliviruses (125).

VLP vaccines are virus-like particles (VLPs) that lack replicative genetic material but enable antigens to be presented in a replicated, ordered sequence similar to that of a virus, boosting immunogenicity (126, 127). Owing to their similarities to native viruses in terms of molecular scaffolds and the lack of genomes, VLPs can effectively trigger both humoral and cell-mediated immune responses without the use of an adjuvant (128). Although in some cases VLPs do not require adjuvant, they typically necessitate the incorporation of adjuvant in the formulation to be immunogenic (18, 101, 129). However, all of this work has yet to be put into practice in a commercial vaccine. Using a recombinant baculovirus that co-expresses the PPRV (Peste des petits ruminants virus) H, N, and M proteins, PPR virus-like particles (VLPs) may be budded from insect cell membranes (130). These VLPs were discovered to elicit powerful virus-specific neutralizing antibodies in mice, suggesting that a VLP-based vaccine candidate for PPR may be created (131). It was demonstrated that the antibodies specific to the F and H proteins were produced in experiments on goats using PPRV VLPs. The goats were three times immunized with 150 or 300 μg VLPs or 10^5^ TCID50 Nigeria 75/1, while the control goats were immunized with either PBS or alum adjuvant alone. After the third immunization, all goats immunized with VLP and PPRV Nigeria 75/1 produced significant levels of antibodies against PPRV F, H, and N proteins, while none of the goats immunized with PBS or alum adjuvant alone exhibited an immunological response. These findings indicated that VLP immunization resulted in substantially higher levels of serum neutralizing antibodies than PPRV Nigeria 75/1 immunization in goats (131). Again, IL-4, IL-10 and IFN-γ were measured in goats after vaccination with 150 or 300 μg VLPs and control goats were treated with PBS or alum adjuvant alone. IL-4, IL-10, and IFN-γ were significantly higher while goats were immunized with 300 μg VLPs than control. Interestingly, IL-4 and IL-10 levels were higher while immunized by PPRV Nigeria 75/1 than the control animals or immunized with VLPs, whereas IFN-γ levels in animals immunized with 300 μg VLPs were substantially lower. IFN-γ concentration in VLP-immunized animal serum were higher, suggesting that VLPs stimulated a cellular immune response in goats. These findings show that VLPs trigger a strong immune response against PPRV infection in small ruminants, suggesting that PPRV VLPs may be used to develop a PPRV vaccine (131).

Bluetongue virus (BTV) VLPs consist of VP3, VP7, VP2, and VP5 proteins. These proteins are expressed in insect cells using baculovirus expression (132–136). A serotype 1, 2, 10, 13, and 17 cocktails with VLPs protected against all five serotypes and partly protected from additional serotype types (137). Large scale sheep experiments with 50–200 sheep each trial demonstrated protection against homologous challenges by the VLP vaccine (138). Despite all these efforts and promising findings, VLPs were not produced in that period. More likely, inactivated BT vaccinations on the market are considerably cheaper and also safe to manufacture (139). Protein and VLP in vegetation production have become more popular and cost-effective alternatives for artificial protein synthesis of complicated high-value proteins (140).

In research, E2 glycoprotein and E^rns^ have been utilized to develop recombinant vaccines to prevent bovine viral diarrhea BVDV illness (98). BVDV-VLPs consisting of dimerized viral proteins E2 and E^rns^, and VLPs consisted of spherical particles of ~50 nm in diameter (141). Mice vaccinated with 15 μg of ISA201-adjuvant VLPs produced increased E2-specific antibodies such as IgG, IgG1, and IgG2a and increased neutralizer activity in BVDV to control (142). Stimulation of splenocytes from VLP-immunized mice resulted in substantially higher numbers of cells of CD3^+^CD4^+^T and CD3^+^CD8^+^T. Furthermore, the proliferation and production of Th1-associated IFN-γ and Th2-associated IL-4 were significantly increased as opposed to the non-stimulated control group of the splenocytes. These results showed that BVDV-VLPs elicited BVDV-specific humoral and cellular immune responses to mice effectively, indicating a promising potential to create BVDV-VLP-based vaccines for BVDV infection prevention (142). Again, a shortened form of E2 glycoprotein (tE2) of BVDV has been expressed in tobacco plants (143). The construct was improved with a signal peptide to guide the protein into the plant secretory route, Kozak consensus sequence, and KDEL retention signal. Recombinant protein accumulated up to 20 μg of tE2 per gram of fresh leaves. Immunization of guinea pigs with 20 μg of tE2 induced neutralizing antibodies comparable to those induced by a whole virus vaccine (143). Transgenic alfalfa plants were created, which express tE2 of BVDV fusion into a single-chain antibody that aims to deliver antigen cells (APCH-tE2) (144). APCH-tE2 was stably expressed, and the antigen accumulation in all the clones tested was comparable. The recombinant vaccination produced high neutralizing antibody titers (145). In addition, the experimental vaccine was assessed with two doses of 3 μg APCH-tE2 injected in animals. The immunogen elicited a robust antibody reaction that was neutralizing (146). More importantly, they demonstrated full virological protection when animals were challenged by virulent BVDV (146).

It was investigated that Foot and mouth disease virus (FMDV) type O/IND/R2/75 polyprotein genes encoded recombinant FMD virus like particles (VLPs) expressed in Sf9 cells and adjuvanted with CpG and Poly I:C induced protective immune responses in guinea pigs *via* FMDV (147, 148). Guinea pigs vaccinated with VLP + CpG had significantly higher cell mediated immunity (CMI) than traditional vaccine groups as evident from higher IgG2 levels than IgG1. Although in VLP + CpG vaccine, humoral response was less than conventional vaccines, but VLP + CpG had a greater lymphocyte stimulation index than conventional VLP and VLP + Poly I:C. The challenge tests with 28 and 56 dpv showed 75% protection in VLP + CpG vaccinated guinea pigs primary and boosted animals, while 50 and 62% protection in primary and boosted animals in VLP + Poly I:C, respectively (149–151). Again, a recombinant baculovirus clone encoding P1-2A-3C coding sequences of foot-and-mouth disease virus (FMDV) serotype O (1) Manisa was generated. FMDV structural proteins were expressed in Sf9 cells together with 3C protease, and the generation of virus-like particles (VLP) was investigated. The recombinant protein was prepared as a vaccine using an oil adjuvant, ISA 206, and the vaccine's potency was evaluated in cattle (152–154). The potency value of the vaccine [PD (50)] was 5.01, and most inoculated animals developed neutralizing antibody titers after two vaccinations. CpG adjuvant in eliciting protection in VLP-based FMD vaccinations was shown to be higher, followed by ISA206 and Poly I:C in guinea pigs (155).

Development of chimeric virus-like particles (VLPs) with FMDV epitopes has shown significant humoral responses in the vaccinated pigs but only limited protection against the homologous challenge. Recombinant adenovirus expressing the highly conserved non-structural FMDV 3D protein and its ability to elicit particular T-cell responses in a mouse model were developed (156). Rangel G also presented two distinct prime-boost methods—FMDV serotype C-specific chimeric VLP and mice immunogenicity analysis (157).

Unfortunately, there is no commercially available ruminant vaccine. Still, in the veterinary field, only porcine circovirus type 2 (PCV2) VLP-based vaccine is commercially available (Porcilis PCV-manufactured by Intervet International, The Netherlands) (158, 159). Some ruminant VLP vaccines are in clinical trials, such as FMDV (152, 155, 157, 160), BTV (161, 162), and RVFV (163).

## DIVA (Differentiating Infected From Vaccinated Animals) Vaccine

Even though effective conventional vaccines are available for a few ruminant viral diseases, they cannot be utilized. Because they would interfere with disease monitoring based on serological testing and may cause a country to lose its disease-free status. A classic example is FMD in sheep. Including the fact that inactivated FMD vaccinations have been around for a long time and are very good at controlling clinical illness (164). They are not used in FMD-free countries because doing so will jeopardize their position and trigger foreign commerce to be disrupted. Conventional vaccinations, on the other hand, also decreased epidemic incidence in enzootic regions, and vaccination was used to monitor the disease's dissemination in a outbreak in the Netherlands (165). The vaccinated livestock, on the other hand, were slaughtered to enable the country to rapidly recover its FMD-free status. The ability to recognize and selectively remove genes from a pathogen has contributed to the creation of “marker vaccines” that, when paired with adequate diagnostic assays, can distinguish infected from vaccinated animals (DIVA) by distinguishing antibody responses induced by the vaccine (no antibodies produced to deleted genes) from those induced during wild-type virus infection. DIVA vaccinations and screening testing are now available or in progress for a variety of diseases, including infectious bovine rhinotracheitis (IBR) and FMD. IBR, which is triggered by BHV-1 infection in livestock, has been listed as a candidate for eradication from national herds around the world, prompting DIVA to improve vaccinations and diagnostics. The need for a marker (DIVA) vaccine for IBR in Europe was fulfilled by the synthesis of a glycoprotein E (gE)-deleted vaccine using traditional methods (166). Although the gE protein is not necessary for viral replication, it is critical for viral intercellular dissemination, especially in nerve cells. Basic diagnostic tests dependent on gE deletion have been developed using both gE-blocking enzyme-linked immunosorbent assay (ELISA) and PCR amplification techniques (167, 168). Because there are serious concerns about the long-term viability of “stamping-out” strategies in regions with high animal population density, there is a lot of money being invested in DIVA vaccines for FMD (169, 170). Since subunit antigen vaccines only offer a limited number of epitopes to the immune system of the species, they have been relatively unsuccessful, and several antigens are normally needed for defense. The aim of current research is to establish responsive tests (ELISA) for antibodies against non-structural proteins and combinations of capsid proteins, such as empty capsid delivered by different expression systems (170). Bluetongue virus in sheep, Peste des petits ruminants, and bovine viral diarrhea are only a few of the diseases for which a DIVA solution is extremely beneficial but still in the works (171–173).

## Vectored Vaccine

The discovery of new prophylactic and therapeutic vaccine candidates has been aided by antigen/gene delivery systems (115). A vector is used to transmit defensive protein(s) to the vaccinated host's immune system in vector vaccine technology (174). A wide variety of vector types, both replicating (Adenovirus, Measles virus, Pox virus-Vaccinia, Vesicular stomatitis virus) and non-replicating (Adenovirus, Alphavirus, Herpesvirus, Pox virus-NYVAC, Pox virus -MVA, Pox virus-ALVAC, and Pox virus-FPV) are available (175). The proper vaccination for a given situation will rely on the biology of the target pathogen, the quantity and amount of gene inserts, and whether the vaccine is to be used to prevent infection or provide protection to those already infected. In certain instances, these vectors are immunogenic and may present several antigens (176).

There are recombinant vaccines in which BTV antigen-specific genes are carried by recombinant viruses and expressed in the host. Since the viral vectors (carrier viruses) used for this reason have been attenuated, they are deemed safe (177). Furthermore, they have BTV antigen-specific genes (transgenes) but neglect the parental BTV's molecular regulatory elements. As a result, the chance of gene segment reassortment with field BTV strains is significantly reduced (178). Viruses that may express the BTV VP2 gene, such as capripox, canarypox, vaccinia, and herpes virus, have been used to make recombinant viral vector vaccines with differing degrees of effectiveness (179, 180). Again, Poxviruses replicate in the cytoplasm of infected cells, effectively eliminating the possibility of genomic incorporation and viral survival in the host DNA. It is also possible to accommodate large pieces of foreign DNA. Any Poxviridae family members, such as canarypox, capripox, and vaccinia viruses, have been used to express BTV antigens (179, 181).

Herpesviruses have a dsDNA genome, which allows for a massive transgene insert scale. Using an equine herpes viruses construct, recombinant-vectored vaccines against BTV8's VP2 and VP5 genes were developed. In experimental vaccination in IFNAR (−/−) mice, these vaccinations only offered limited safety during the challenge (182). Similarly, the BTV VP2 gene was expressed in a non-pathogenic bovine herpes virus 4 (BoHV4) strain, and recombinant BoHV4 -VP2 construct was created. The experimental model- the IFNAR (−/−) mouse, showed limited protection against the BoHV4 -VP2 construct (183).

Recombinant adenovirus vectors are being used as vaccine candidates for a variety of viral diseases since they can trigger T cell immunity. Replication-defective recombinant human adenovirus serotype 5 (Ad5) expressing VP2, VP7, or NS3 BTV proteins were given to IFNAR(–/–) mice and sheep. As evidenced by humoral and cellular immune responses (BTV-specific CD8+ and CD4+ T cells), mice vaccinated with different rAd5 showed full protection against BTV challenge (184). Sheep had mild disease signs and lower viremia after vaccination with Ad5-BTV-VP2 and Ad5-BTV-VP7, or only with Ad5-BTV-VP7 followed by BTV challenge. Sheep were inoculated Ad5-BTV-VP7 formed ample BTV-specific CD8+ T cells but there were no neutralizing antibodies (185).

Another study used the VP7 core protein of BTV2 to cause an immune response in sheep using a non-replicative canine adenovirus type 2 (Cav-VP7 R0) or a leporipoxvirus (SG33-VP7). Both recombinant antigens elicited a humoral immune response in cattle. Only Cav-VP7 R0 elicited an important antigen-specific CD8+ cell response, whereas both SG33-VP7 and Cav-VP7 R0 elicited an adequate antigen-specific CD4+ response. Sheep given the Cav-VP7 R0 vaccine is later exposed to either homologous serotype BTV2 or heterologous serotype BTV8. As determined by real-time PCR in plasma, the immune response caused by Cav-VP7 R0 was insufficient to provide protective immunity against BTV (Bouet-Cararo) (186). It only gave homologous serotypes partial immunity. It implies that the function of BTV core proteins in cross-protective immune responses should be explored further (186).

Animals, including sheep, goats, and cattle, were protected against a fatal challenge by receiving a single dose of a recombinant adenovirus expressing the surface glycoproteins of Rift Valley fever virus (RVFV) (187). Sheep were vaccinated two recombinant replication-defective human adenoviruses serotype 5 (Ad5) expressing either the highly immunogenic fusion protein (F) or hemagglutinin protein (H) from Peste des petits ruminants virus (PPRV) by intramuscular inoculation. PPRV-specific B- and T-cell responses were induced by both recombinant adenovirus vaccinations. As a result, neutralizing antibodies were detected in serum from vaccinated sheep (188). In 2012, the United States Department of Agriculture (USDA) approved conditional licensing to the Adt.A24 FMD vaccination to protect cattle. The replication deficient Adt.A24 vaccine uses a human adenovirus construct as a vector to deliver empty capsids of A24 FMD strain to induce an immune response (189). Previous investigations in bovine have demonstrated that the Adt.A24 vaccine prevents FMD and FMD viremia 7 days after first immunization, and combined with the ENABL^®^ adjuvant being the most effective (190, 191). This vaccine does not have a tendency to become virulent again, it doesn't shed from vaccinated animals to naïve ones, and it doesn't provide a dairy cow the ability to excrete the vaccine *via* her milk, thus it has a 64% effectiveness rate in clinical FMD (192, 193).

## DNA and RNA Vaccine

Antigen production is induced in the host by DNA vaccines. A plasmid containing a viral gene that can be expressed in mammalian cells or a gene encoding a mammalian protein that can be expressed in mammalian cells is known as a DNA (or RNA) vaccine (121). The requisite genetic elements, such as solid eukaryotic promoters for transcriptional control, a polyadenylation signal sequence for stable and effective translation, and a bacterial replication origin, are integrated into a plasmid (194, 195). The plasmid is transfected into host cells and transcribed into mRNA, which is then encoded, causing the host cellular machinery to produce an antigenic protein (196). The host immune system recognizes the expressed proteins as foreign, resulting in the formation of cellular and humoral immune responses (197, 198). Immunizing animals with naked DNA encoding protective viral antigens would be useful for viral vaccines in some situations (199) because it overcomes the safety problems associated with live vaccines and vector immunity while still allowing cytotoxic T cells to trigger and express the antigens intracellularly (120, 200).

Early studies propose using a gene producing the VP4 protein of bovine rotavirus (BRV), which has been shown to be efficient in generating a Th1-like immune response, to combat viral infections in cattle (201). Later, it was found that the plasmid expressing the envelope glycoprotein gp51 and transmembrane glycoprotein gp30 of the bovine leukemia virus (BLV) could generate an efficient cellular immune response (202). Furthermore, researchers have discovered that the DNA vaccine encoding fusion (F) gene from bovine respiratory syncytial virus (BRSV) is capable of providing calf immunity against the disease (203). It was reported that DNA immunization with gC gene of bovine herpes virus-1 (BHV-1) may lead to neutralization antibody and lympho-proliferative responses in cattle (204, 205). Furthermore, BHV-1 gB and IL-12 have been proposed to improve CTL responses. Plasmid-based suppositories that include the gD gene of BHV-1 help promote mucosal immunity and also improve the immune responses of bovine CD 154 co-stimulatory molecules connected to the gD gene (206, 207). Additionally, it has been suggested that the gD gene is more protective than the gC gene. In addition, the capacity of BHV-1 VP22 protein to affect intracellular trafficking has been used to enhance the effectiveness of a DNA vaccine expressing gD gene (208). Another nucleic acid vaccine is now available that is expected to prevent cattle from contracting bovine viral diarrhea virus (BVDV). BVDV type 1 glycoprotein E2-expressing plasmid DNA elicited virus-specific neutralizing antibodies (209). One possible approach to BVD vaccine development is using the non-structural protein NS3 to promote humoral protection (210). It was reported that compared to the administration of DNA or protein vaccines alone, the DNA prime boost regimens were efficacious in the prevention of BVD in cattle (211). The development of vaccinations for foot and mouth disease is being advanced through the use of VP1-based DNA vaccines (113, 212). Plasmid DNA encoding the FMDV VP1 protein, followed by boosting with a VP1 peptide conjugate, resulted in high titers of neutralizing antibodies, indicating that the prime-boost approach may be a critical component in the development of a DNA vaccine against FMD (213). A microparticulate-based DNA vaccine that codes for the T and B cell epitopes of the FMDV's VP1 has recently been produced (214).

## Vaccine Delivery Systems

A successful vaccination is dependent on effective vaccine administration. Most vaccinations are injected into the body through subcutaneous (SC) or intramuscular (IM) routes. The delivery of a hypodermic injection is linked with suffering and agony, which may lead to a patient's non-compliance and the need for specialized staff. They are linked to the spread of disease owing to the danger of needle-stick injuries or re-use of infected needles. Problems may arise when mass immunization is required because of insufficient vaccine availability or limitations in vaccine manufacturing (215, 216). Most vaccinations now are administered into the subcutaneous fat or under the skin's muscle. Only a little amount of vaccinations are injected into the viable skin (epidermis and dermis) (217–219). Dendritic cells (DCs) in the tissues take up the antigen, digest it, and deliver it to T lymphocytes in the draining lymphoid organs *via* each of these routes of application. While DCs are sparse in subcutaneous fat and muscular tissue, the dermis and epidermis are heavily inhabited by various subsets of DCs. Therefore, by using hypodermic injection, antigen delivery will avoid the skin's immune cells, which will result in a less effective vaccination. As a result, the skin is an ideal site for vaccine administration since vaccination at this site will evoke strong immune responses at much lower doses of antigen than intramuscular vaccine (220). Recent research shows that the nasal mucosa and the gastrointestinal system may potentially be good sites for vaccine delivery (221, 222). These alternative routes of delivery have the potential to elicit immune responses that are qualitatively different from those elicited by injected vaccines, or to stimulate immune responses at these mucosal sites, allowing for more effective defense against pathogens that enter through these routes e.g., oral or nasal (18, 223). Disabled infectious single cycle (DISC) viruses use alternative routes of delivery and these routes have the potential to elicit immune responses that are qualitatively different from those elicited by injected vaccines, or to stimulate immune responses at these mucosal sites, allowing for more effective defense against pathogens that enter through these routes (224).

## Molecular Biology Techniques and Bioinformatics Analysis Are Being Used in the Production of Next Generation Vaccines

Pathogen genomic research and increased knowledge of pathogenesis pathways have led to the discovery of new antigens and the creation of recombinant veterinary vaccines. Viruses, prokaryotes, and eukaryotes viruses have also been exposed to whole-genome and draft sequencing (225, 226). These advancements have also improved antigen discovery and heterogeneity classification amongst viral pathogens, which generally have fewer than 10 genes, and eukaryotic pathogens, which typically have >10,000 genes (227–229). Since relevant antigenic structures may identify and produce recombinant vaccines containing only the antigen needed to elicit protective immunity, genome sequencing technologies, and methods for screening a pathogen's genome and proteome have greatly improved antigen discovery performance. Whole-genome fragments and the whole repertoire of encoding proteins are often used in genomic repositories, allowing for vaccine screening (106, 230).

Genetic sequencing and bioinformatics have led to a huge quantity of genetic data on pathogens and their characteristics. To match phenotypic characteristics to their genetic origin, reverse genetics (RG) may be utilized. Genetic perturbations are introduced into a gene of interest using different methods, and the effects are studied utilizing phenotypic and functional studies. In recent years, the technologies used in RG have quickly developed from traditional techniques to the utilization of clustered regularly interspaced short palindromic repeat (CRISPR)/associated protein 9 (Cas9) technology, which is revolutionizing genome editing procedures. RG has contributed to the understanding of viral replication, transcription and translation, assembly and budding, virus–host cell protein interactions, identification and characterization of viral fitness determinants, and investigation of the mechanisms by which viruses counter host antiviral defenses. In the field of vaccine production, recombinant DNA techniques and RG have allowed targeted genetic changes in viral genomes aimed at attenuating or neutralizing viral pathogenicity, as well as producing DIVA vaccines to aid disease monitoring and epidemiological research. Infectious clones of the transmissible gastroenteritis virus were developed using RG and were able to produce immunity in infectious bovine rhinotracheitis (231). RG has also been used to develop vaccines against FMD (232) and the bluetongue virus (233). Another use of RG is to create viruses that lack a critical gene and therefore cannot replicate themselves in vaccinated hosts, known as disabled infectious single cycle (DISC) viruses (234). These viruses, upon entrance into a host cell, can reproduce just once, triggering the host's immune response and making traditional infection impossible. The strategy has been used in the development of bluetongue virus vaccines (224).

A reverse vaccinology (RV) method uses algorithms to analyze the whole genome of pathogens, synthesizes their protein and peptide antigens, and then performs *in vitro* research on them prior to doing *in vivo* investigations (230, 235, 236). This program and many others that identify T-cell and B-cell epitopes all need a central software package, namely, epitope mapping software (237–240). Vaxign is a web-based software for mapping both MHC class I and class II restricted antigens that is publicly accessible (www.violinet.org/vaxign/) (241). Other web-based software that predicts MHC-I (http://tools.iedb.org/mhci) and MHC–II (http://tools.iedb.org/mhcii) epitopes are also publicly available (242). A total of 5–15 years of development time was required for traditional vaccination methods, while the RV method has reduced that time to 1–3 years. In an early stage study, vaccine candidates were developed using RV for Histophilus somni which is associated with the bovine respiratory disease (BRD) complex and for which the available vaccines are suboptimal (243).

## Conclusion

Infectious diseases will continue to pose a major challenge to the global economy and public health in the years ahead, as shown by the spread of microbial pathogens such as FMDV, PPRV, and BTV, as well as the emergence of drug-resistant pathogens and the threat to bioterrorism. Ruminant vaccine development programs have been given high priority due to the huge economic losses caused by various ruminant diseases. More oriented approaches for developing more protective vaccines have benefited from the advent in molecular genetics and better understanding of infectious disease immunobiology. Identification of virulence factors and immunogenic antigens has been important in the production of new vaccine generations, which has been augmented by rapid advances in recombinant vectors. A combination of these factors will almost certainly allow for the Development and production of vaccines that are less costly, more potent, safer, and simpler to administer.

## Author Contributions

All authors made substantial contributions to conception and design, acquisition of data, analysis and interpretation of data, took part in drafting the article or revising it critically for important intellectual content, agreed to submit to the current journal, gave final approval of the version to be published, and agree to be accountable for all aspects of the work.

## Funding

This work was supported by a grant from the National Natural Sciences Foundation of China (no. 31602037) and the Key Technologies R&D Program of Gansu Province (19ZDNA001).

## Conflict of Interest

The authors declare that the research was conducted in the absence of any commercial or financial relationships that could be construed as a potential conflict of interest.

## Publisher's Note

All claims expressed in this article are solely those of the authors and do not necessarily represent those of their affiliated organizations, or those of the publisher, the editors and the reviewers. Any product that may be evaluated in this article, or claim that may be made by its manufacturer, is not guaranteed or endorsed by the publisher.
